# Supplementary material for: De novo identification of microbial contaminants in low microbial biomass microbiomes with Squeegee
Source: Nat Commun. 2022 Nov 10;13:6799. doi: 10.1038/s41467-022-34409-z (PMC9649624; doi:10.1038/s41467-022-34409-z)
Supplement: Supplementary file 1 — Supplementary Information [file 41467_2022_34409_MOESM1_ESM.pdf]

1 Benchmark with Decontam using the strict ground truth

Figure 2b shows the precision, recall, and F-score of Squeegee and Decontam at both species and genus rank using the strict ground truth set. At species rank, Squeegee achieved an unweighted precision of 0.643 (9/14 species) and an unweighted recall of 0.600 (9/15 species). The false positive calls for Squeegee are: *Rothia mucilaginosa*, *Staphylococcus cohnii*, *Staphylococcus mitis*, *Staphylococcus haemolyticus*, and *Escherichia coli*. The unweighted F-score for Squeegee is 0.621. On this dataset, Decontam achieved an unweighted precision of 0.053, an unweighted recall of 0.600, and an unweighted F-score of 0.097.

We also evaluated both methods with weighted scores, taking into account abundance information. Each of the species are weighted by the mean fraction of reads assigned to those species in the non-negative samples. The weighted precision, weighted recall, and weighted F-score for Squeegee is 0.502, 0.835, and 0.627, and for Decontam is 0.651, 0.459, and 0.538. We also took a closer look at the predicted contaminants output by each of the methods, and their relative abundance in the negative control samples. The 9 correctly predicted species by Squeegee occupy over 0.835 of the cumulative relative abundance from the composition of the ground truth contaminants. Using the same measurement, Decontam identifies 0.616 of the cumulative relative abundance from the ground truth species.

At genus rank, while evaluating using the strict ground truth set, Squeegee achieved an unweighted precision of 0.750 (9/12 genera) and an unweighted recall of 0.692 (9/13 genera), and resulted in an unweighted F-score of 0.720. Decontam achieved an unweighted precision of 0.145, an unweighted recall of 0.769, and an unweighted F-score of 0.244. When evaluating both methods by weighting each genus by the mean fraction of reads of all genera found in the non-negative samples. The weighted precision, weighted recall, and weighted F-score for Squeegee was 0.391, 0.897, and 0.545, respectively, and the weighted precision, recall, and F-score of Decontam was 0.800, 0.773, and 0.787, respectively. When evaluated with recall weighted by the relative abundance of the predicted genera in the negative control samples, both methods performed well, with Squeegee scored at 0.940 and Decontam scored at 0.922. Although Decontam mislabeled some of the high abundance contaminants at species rank, it did label some of the closely related species under the same genera as contaminants, resulting in a significant increase of the score at genus rank.

Supplementary Table 1. Parameters and Dataset Characteristics

	Simulated	Maternal/infant	HMP	Human RNA-Seq
Total number of samples	126	344	749	40
Total number of sample types	7	9	16	6
Prevalence min read	30	30	30	30
Prevalence min abundance	0.05%	0.05%	0.05%	0.05%
Min genome coverage	7.5%	2.5%	20%	1.0%
Min combined score	0.75	0.75	0.75	0.75
# of negative control experiment samples	N/A	10	N/A	N/A
# of ground truth species	12	strict/permissive: 15/31	N/A	N/A
# of ground truth genus	9	strict/permissive: 13/16	61	N/A
# of predicted species	0.25%/0.5%/1%: 5/6/8	14	75	7
# of predicted genus	0.25%/0.5%/1%: 3/4/6	12	24	6
# of correct predicted species	0.25%/0.5%/1%: 5/6/8	strict/permissive: 9/10	61	N/A
# of correct predicted genus	0.25%/0.5%/1%: 3/4/6	strict/permissive: 9/10	16	N/A

Supplementary Table 2. Genera presence/absence across different body sites.

	Vaginal	Throat	Stool	Oral	Skin	Nasal
Alistipes			✓			
Bacteroides			✓			
Campylobacter		✓				
Corynebacterium						✓
Cutibacterium					✓	✓
Fusobacterium		✓		✓		
Haemophilus		✓		✓		
Lactobacillus	✓					
Neisseria		✓		✓		
Prevotella		✓		✓		
Parabacteroides			✓			
Phocaeicola			✓			
Rothia				✓		
Staphylococcus					✓	✓
Streptococcus		✓		✓		
Veillonella		✓		✓		
Xanthomonas	✓				✓	✓

Supplementary Table 3. Contaminant Score Calculation

	Calculation
Prevalence Score	For each sample type, calculate the prevalence rate $p_{i,t}$ of species i in sample type t as:<br><br>$p_{i,t} = \frac{\text{occurrence of } i \text{ in sample type } t}{\text{sample count of } t}$<br><br>Then calculate mean prevalence for i as:<br><br>$P_i = \frac{\sum_{t=1}^{T} p_{i,t}}{T}$, where T is the total number of sample types.
Mash Score	For each sample type, calculate the paired-wise mash distance m_i of species i for all samples that species exist.<br>Then calculate mash score for i as:<br><br>$M_i = \text{mean of } m$,<br><br>where m is a subset of m_i such that element in m is above 90th percentile of all m_i
Alignment Score	For each sample type, calculate the breadth of genome coverage $a_{i,t}$ of species i in sample type t as:<br><br>$a_{i,t} = \frac{\text{length of } G_i \text{ with at least 3 reads covered in sample type } t}{\text{total length of } G_i}$,<br><br>where G_i is the reference genome of species i.<br>Then calculate alignment score for i as:<br><br>$A_i = \frac{\sum_{t=1}^{T} a_{i,t}}{T}$, where T is the total number of sample types.
Combined Score	The combined score is calculated by normalizing each of the score using the equation below:<br><br>$C_i = \frac{1}{3} \cdot (\frac{P_i}{\overline{P_i}} + \frac{M_i}{\overline{M_i}} + \min(\frac{A_i}{5 \cdot \text{min\_cov}}, 1))$<br><br>where $\overline{P_i}$ is the mean of prevalence score of all candidate species, and $\overline{M_i}$ is the mean of mash score of all candidate species, and min_cov is the minimum coverage threshold defined by the user.

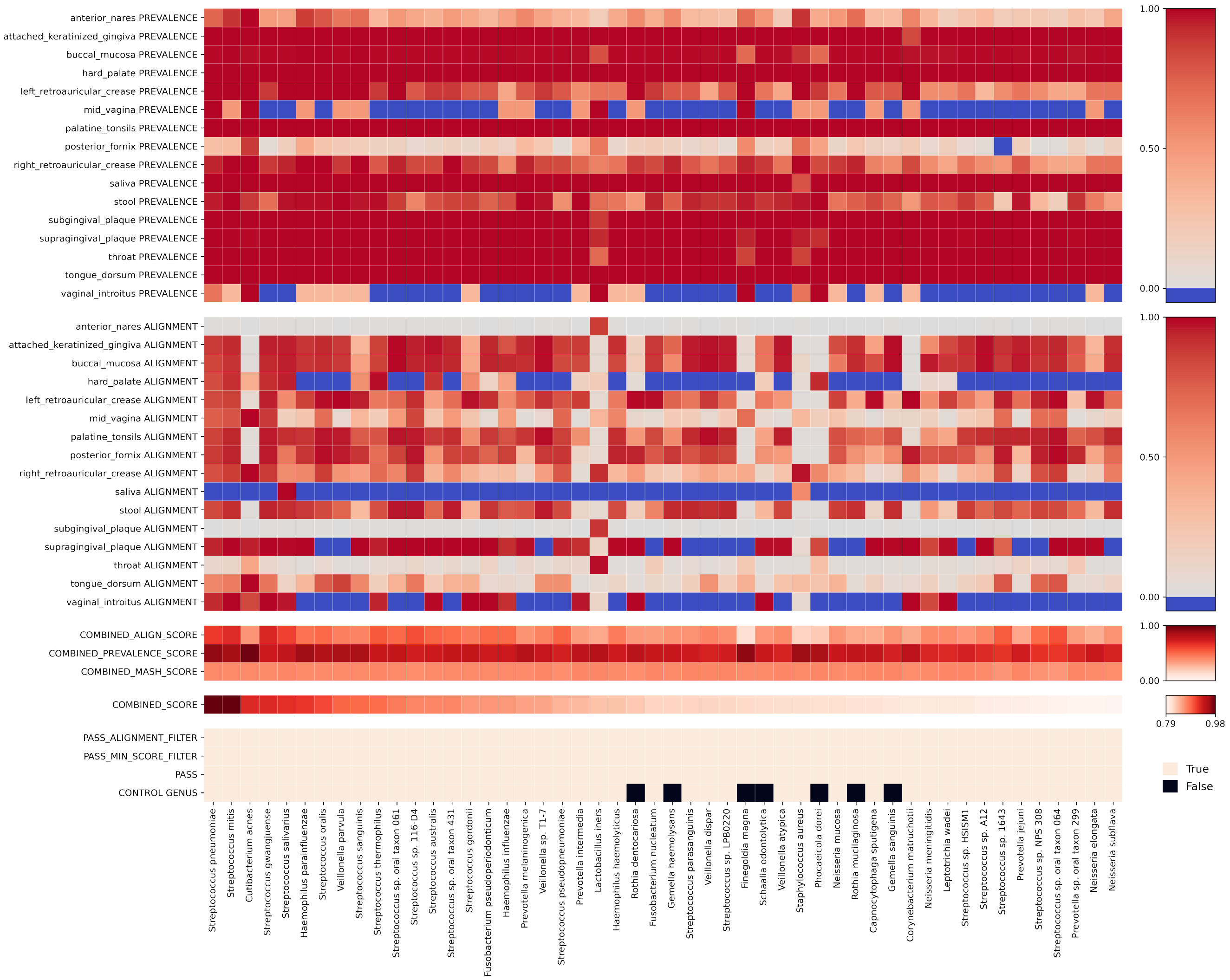

Supplementary Figure 1. Scoring and filtering of candidate contaminants for the HMP dataset. This plot shows the prevalence, the breadth of genome coverage, and additional score and filtering information of the top 50 contaminant species after filtering. The first 16 rows show the prevalence of each species among each of the sample types, where zero prevalence is marked in blue. The next 16 rows show the breadth of genome coverage of each species in each of the sample types, where zero is marked in blue. The remaining rows show the prevalence score, the alignment score, the Mash score, and the combined score used to make the final prediction, and whether each species passes the filters. The last row of the heat map shows whether the species can be found in the ground truth with true positive show in white and false positive show in black. Source data are provided as a Source Data file.

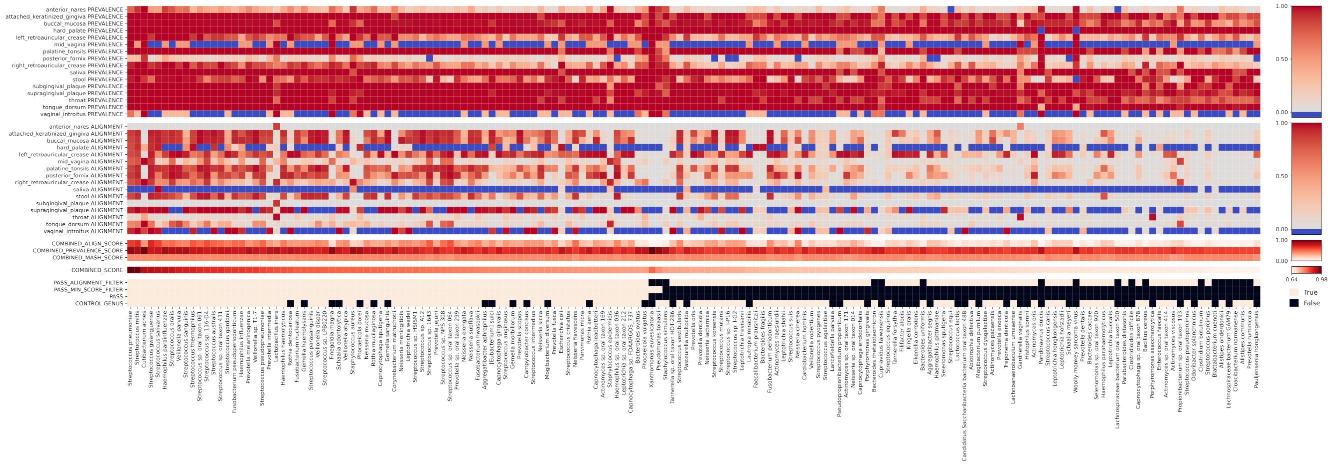

Supplementary Figure 2. Scoring and filtering of candidate contaminants for the HMP dataset. This plot shows the prevalence, the breadth of genome coverage, and additional score and filtering information of candidate predicted contaminant species. The first 16 rows show the prevalence of each species among each of the sample types, where zero prevalence is marked in blue. The next 16 rows show the breadth of genome coverage of each species in each of the sample type, where zero is marked in blue. The remaining rows show the prevalence score, the alignment score, the Mash score, and the combined score used to make the final prediction, and whether each species passes the filters. The last row of the heat map shows whether the species can be found in the ground truth with true positive show in white and false positive show in black. Source data are provided as a Source Data file.

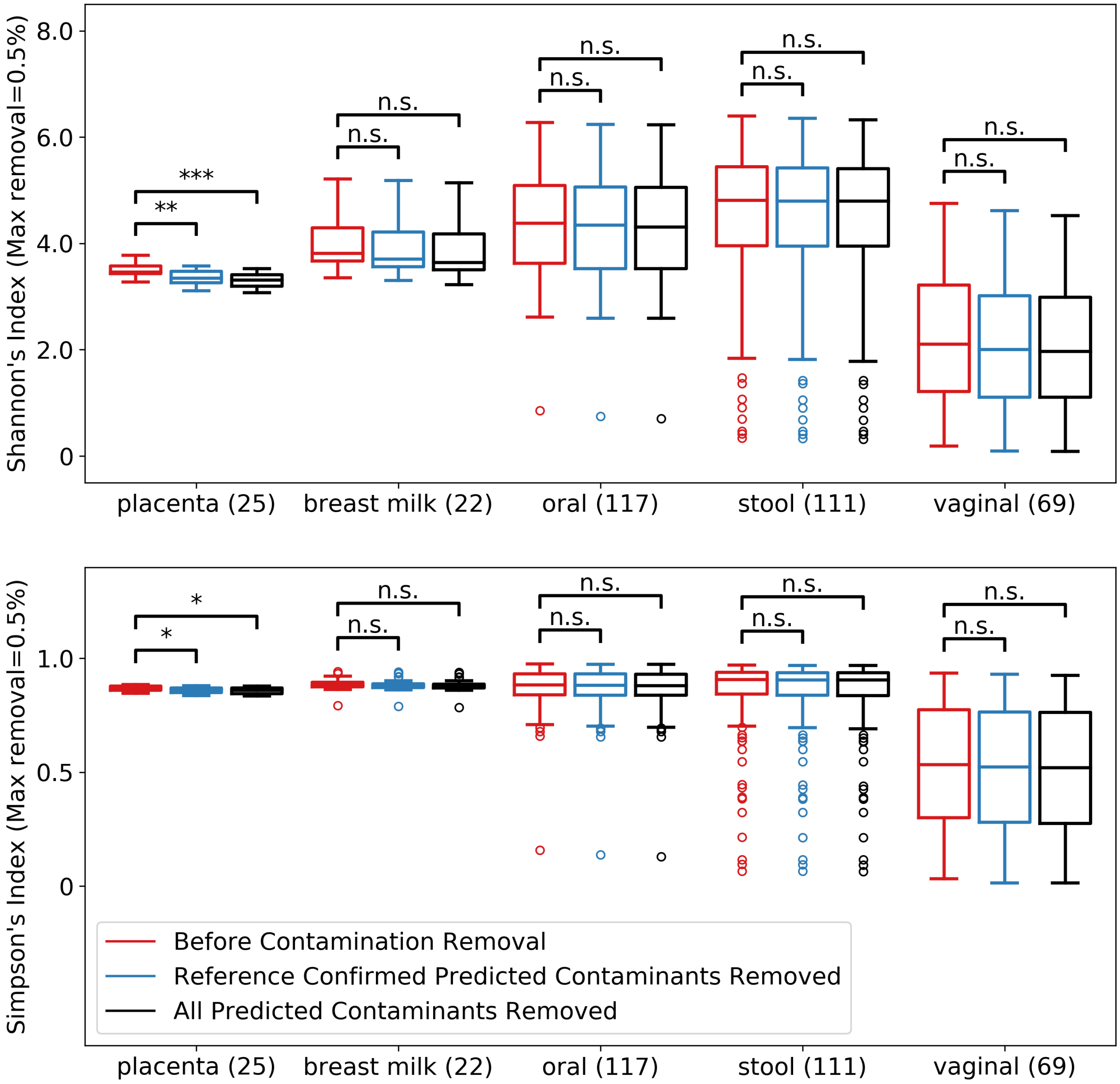

Supplementary Figure 3. Alpha diversity index for maternal/infant dataset. Both Shannon's and Simpson's diversity index of the communities in each of the samples were evaluated before the contaminant reads were removed (red), after removing species only confirmed by the experimental negative control (blue), and after removing all species predicted by Squeegee (black). The max removal is set to 0.5%. Numbers inside parentheses are the numbers of samples in each sample type. The significance test was done using two-sided Mann–Whitney U test for all combined sample types with more than 20 samples. No adjustments were made for multiple comparisons. Significance labeling: n.s.($P>0.05$), *($P\leq0.05$), **($P\leq0.01$), ***($P\leq0.001$). Each box plot includes the median line, and the box bounds the interquartile range (IQR). The Tukey-style whiskers extend from the box by at most 1.5 × IQR. The circle denotes outliers that extend beyond the whiskers. The exact p-value between Shannon's index before removal and reference confirmed contaminants removed is $1.2\text{x}10^{-3}$ for placenta samples. The exact p-value between Shannon's index before removal and all contaminants removed is $7.6\text{x}10^{-5}$ for placenta samples. The exact p-value between Simpson's index before removal and reference confirmed contaminants removed is $1.4\text{x}10^{-2}$ for placenta samples. The exact p-value between Simpson's index before removal and all contaminants removed is $4.2\text{x}10^{-2}$ for placenta samples. Source data are provided as a Source Data file.

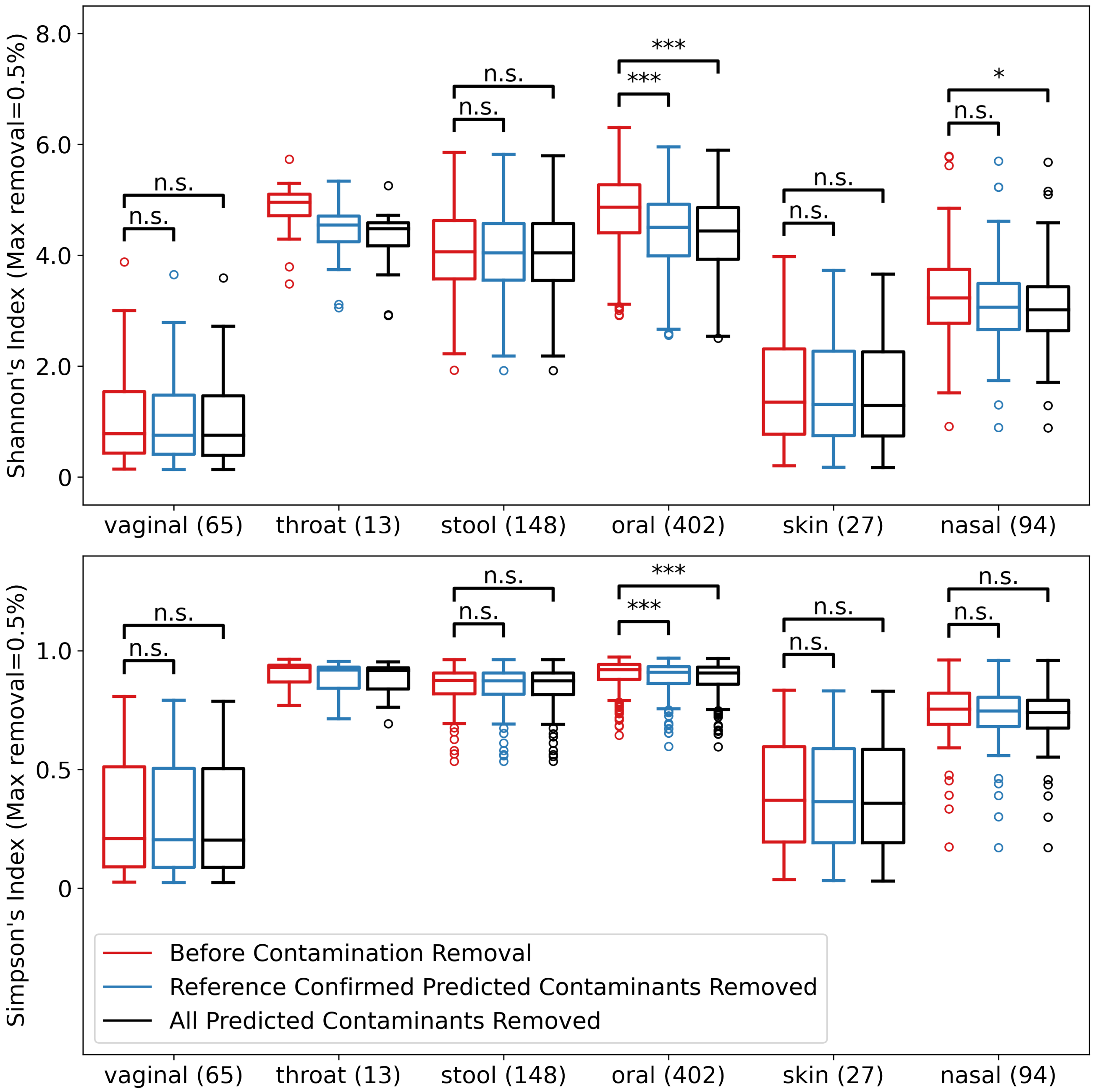

Supplementary Figure 4. Alpha diversity index for HMP dataset. Both Shannon's and Simpson's diversity index of the communities in each of the samples were evaluated before the contaminant reads were removed (red), after removing species only confirmed by the experimental negative control (blue), and after removing all species predicted by Squeegee (black). The max removal is set to 0.5%. Numbers inside parentheses are the numbers of samples in each sample type. The significance test was done using two-sided Mann–Whitney U test for all combined sample types with more than 20 samples. No adjustments were made for multiple comparisons. Significance labeling: n.s.(P>0.05), *(P≤0.05), ***(P≤0.001). Each box plot includes the median line, and the box bounds the interquartile range (IQR). The Tukey-style whiskers extend from the box by at most 1.5 × IQR. The circle denotes outliers that extend beyond the whiskers. The exact p-value between Shannon's index before removal and reference confirmed contaminants removed is 1.3×10^{-14} for oral samples. The exact p-value between Shannon's index before removal and all contaminants removed is 1.1×10^{-19} for oral samples, and 1.6×10^{-2} for nasal samples. The exact p-value between Simpson's index before removal and reference confirmed contaminants removed is 1.2×10^{-4} for oral samples. The exact p-value between Simpson's index before removal and all contaminants removed is 4.7×10^{-6} for oral samples. Source data are provided as a Source Data file.

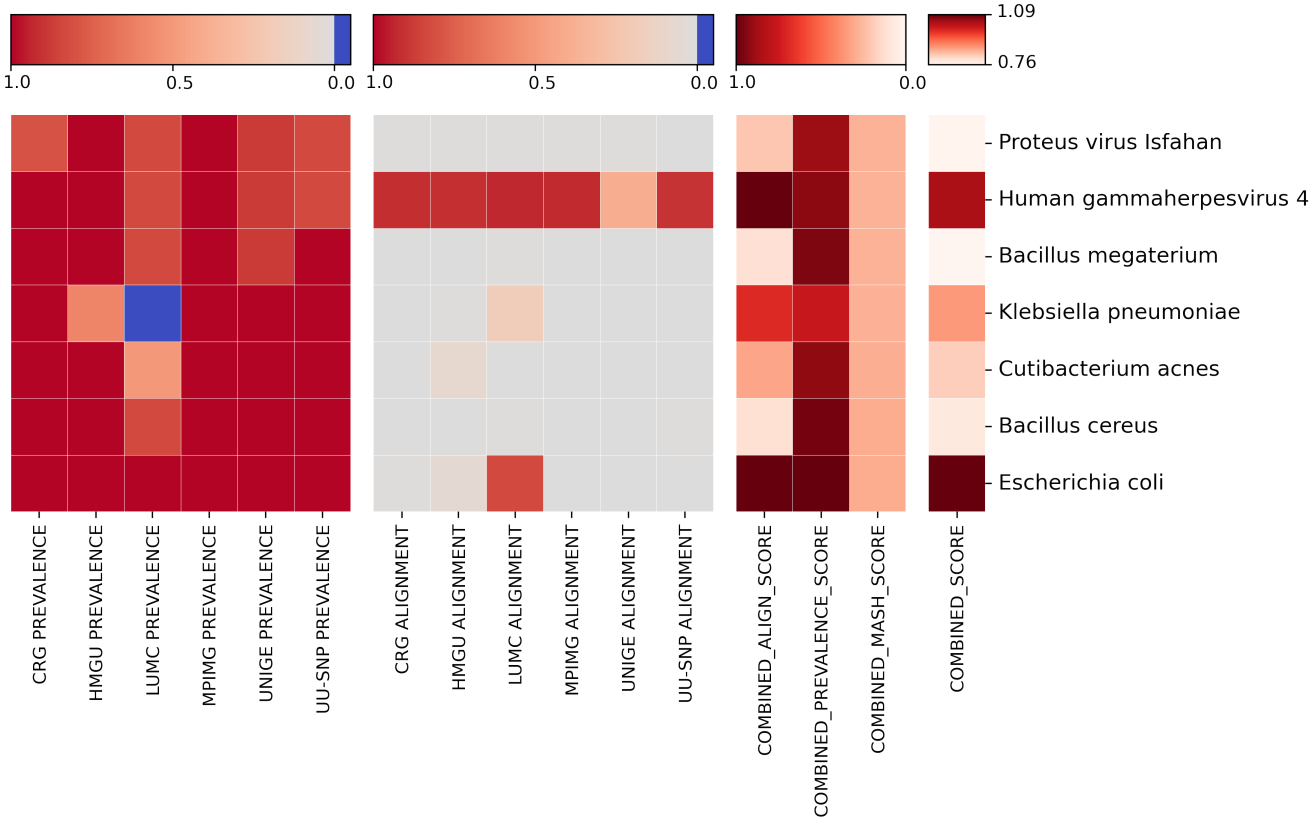

Supplementary Figure 5. Predicted contaminant species for the human RNA-Seq dataset. This figure shows the prevalence, the breadth of genome coverage, and additional scoring information of predicted contaminant species. The first 6 columns show the prevalence of each species among each of the sequencing labs, where zero prevalence is marked in blue. The next 6 columns show the breadth of genome coverage of each species in each of the sequencing labs, where zero is marked in blue. The remaining rows show the prevalence score, the alignment score, the Mash score, and the combined score used to make the final prediction. Source data are provided as a Source Data file.
