# Supplementary material for: De novo identification of microbial contaminants in low microbial biomass microbiomes with Squeegee
Source: Nat Commun. 2022 Nov 10;13:6799. doi: 10.1038/s41467-022-34409-z (PMC9649624; doi:10.1038/s41467-022-34409-z)
Supplement: Supplementary file 2 — Reporting Summary [file 41467_2022_34409_MOESM2_ESM.pdf]

nature research

Corresponding author(s): Todd J. Treangen

Last updated by author(s): Oct 24, 2022

Reporting Summary

Nature Research wishes to improve the reproducibility of the work that we publish. This form provides structure for consistency and transparency in reporting. For further information on Nature Research policies, see our Editorial Policies and the Editorial Policy Checklist.

Statistics

For all statistical analyses, confirm that the following items are present in the figure legend, table legend, main text, or Methods section.

n/a	Confirmed	
☐	☒	The exact sample size (*n*) for each experimental group/condition, given as a discrete number and unit of measurement
☐	☒	A statement on whether measurements were taken from distinct samples or whether the same sample was measured repeatedly
☐	☒	The statistical test(s) used AND whether they are one- or two-sided<br>*Only common tests should be described solely by name; describe more complex techniques in the Methods section.*
☒	☐	A description of all covariates tested
☒	☐	A description of any assumptions or corrections, such as tests of normality and adjustment for multiple comparisons
☐	☒	A full description of the statistical parameters including central tendency (e.g. means) or other basic estimates (e.g. regression coefficient) AND variation (e.g. standard deviation) or associated estimates of uncertainty (e.g. confidence intervals)
☐	☒	For null hypothesis testing, the test statistic (e.g. *F*, *t*, *r*) with confidence intervals, effect sizes, degrees of freedom and *P* value noted<br>*Give P values as exact values whenever suitable.*
☒	☐	For Bayesian analysis, information on the choice of priors and Markov chain Monte Carlo settings
☐	☒	For hierarchical and complex designs, identification of the appropriate level for tests and full reporting of outcomes
☒	☐	Estimates of effect sizes (e.g. Cohen's *d*, Pearson's *r*), indicating how they were calculated

Our web collection on statistics for biologists contains articles on many of the points above.

Software and code

Policy information about availability of computer code

Data collection	Simulated data in this study is simulated with CAMISIM. Non-simulated data in this study can be downloaded from NCBI SRA and NIH Human Microbiome Project website.
Data analysis	Data is analyzed using customized code. The code is available on gitlab: https://gitlab.com/treangenlab/squeegee.git<br>The version of the code we used in this study is also archived at: https://doi.org/10.5281/zenodo.7222151

For manuscripts utilizing custom algorithms or software that are central to the research but not yet described in published literature, software must be made available to editors and reviewers. We strongly encourage code deposition in a community repository (e.g. GitHub). See the Nature Research guidelines for submitting code & software for further information.

Data

Policy information about availability of data

All manuscripts must include a data availability statement. This statement should provide the following information, where applicable:

- Accession codes, unique identifiers, or web links for publicly available datasets
- A list of figures that have associated raw data
- A description of any restrictions on data availability

- The simulated dataset is publicly available and can be downloaded at
https://doi.org/10.5281/zenodo.7064705, https://doi.org/10.5281/zenodo.7062953, and https://doi.org/10.5281/zenodo.7064599. The maternal/infant metagenomic datasets are available for download via NCBI BioProject PRJNA725597. The HMP samples are downloaded from https://www.hmpdacc.org/HMASM/. The human RNA-Seq datasets are available for download via NCBI BioProject PRJEB2123. The datasets were simulated/sequenced from distinct samples, and no sample was simulated/sequenced repeatedly.
- Figures associated with raw data: Figure 2, Figure 3, Figure 4, Figure 5, Figure 6. Supplementary Figure 1, Supplementary Figure 2, Supplementary Figure 3,

Supplementary Figure 4, Supplementary Figure 5
- All sequence dataset that support the findings of this study is publicly available.

Field-specific reporting

Please select the one below that is the best fit for your research. If you are not sure, read the appropriate sections before making your selection.

☒ Life sciences ☐ Behavioural & social sciences ☐ Ecological, evolutionary & environmental sciences

For a reference copy of the document with all sections, see nature.com/documents/nr-reporting-summary-flat.pdf

Life sciences study design

All studies must disclose on these points even when the disclosure is negative.

Sample size	Simulated Dataset: 126 simulated metagenomic samples.<br>Real dataset "maternal/infant": 344 metagenomic shotgun sequencing samples with 10 negative control samples.<br>Real dataset "HMP": 749 metagenomic shotgun sequencing samples from Human Microbiome Project.<br>Real dataset "Human RNA-Seq": 51 human RNA-Seq sequencing runs.
Data exclusions	11 sequencing runs from the Human RNA-Seq dataset is excluded since those runs are sequenced with non-Illumina platform.
Replication	All the experiments are replicated at least twice.
Randomization	The samples are allocated into groups based on the environment (for example, different body sites) the samples are collected from.<br>Randomization is not applicable for this study since the software is designed to search organisms that are shared across multiple groups.
Blinding	Researchers are not blinded during data collection and analysis, because the identification of the group is used as an input for the software to predict contaminant species.

Reporting for specific materials, systems and methods

We require information from authors about some types of materials, experimental systems and methods used in many studies. Here, indicate whether each material, system or method listed is relevant to your study. If you are not sure if a list item applies to your research, read the appropriate section before selecting a response.

Materials & experimental systems

n/a	Involved in the study	
☒	☐	Antibodies
☒	☐	Eukaryotic cell lines
☒	☐	Palaeontology and archaeology
☒	☐	Animals and other organisms
☒	☐	Human research participants
☒	☐	Clinical data
☒	☐	Dual use research of concern

Methods

n/a	Involved in the study	
☒	☐	ChIP-seq
☒	☐	Flow cytometry
☒	☐	MRI-based neuroimaging
